# HIV-1 sub-type C chimaeric VLPs boost cellular immune responses in mice

**DOI:** 10.1186/1476-8518-8-7

**Published:** 2010-11-19

**Authors:** Sirika Pillay, Enid G Shephard, Ann E Meyers, Anna-Lise Williamson, Edward P Rybicki

**Affiliations:** 1 Department of Molecular and Cell Biology, Faculty of Science, University of Cape Town, University Ave, Rondebosch 7701, South Africa; 2 Institute of Infectious Diseases and Molecular Medicine, Faculty of Health Sciences, University of Cape Town, Anzio Rd, Observatory 7925, South Africa; 3 Department of Medicine, Faculty of Health Sciences, University of Cape Town, Anzio Rd, Observatory 7925, South Africa; 4 National Health Laboratory Service, Groote Schuur Hospital, Main Rd, Observatory 7925, South Africa

## Abstract

Several approaches have been explored to eradicate HIV; however, a multigene vaccine appears to be the best option, given their proven potential to elicit broad, effective responses in animal models. The Pr55^Gag ^protein is an excellent vaccine candidate in its own right, given that it can assemble into large, enveloped, virus-like particles (VLPs) which are highly immunogenic, and can moreover be used as a scaffold for the presentation of other large non-structural HIV antigens. In this study, we evaluated the potential of two novel chimaeric HIV-1 Pr55^Gag^-based VLP constructs - C-terminal fusions with reverse transcriptase and a Tat::Nef fusion protein, designated GagRT and GagTN respectively - to enhance a cellular response in mice when used as boost components in two types of heterologous prime-boost vaccine strategies. A vaccine regimen consisting of a DNA prime and chimaeric HIV-1 VLP boosts in mice induced strong, broad cellular immune responses at an optimum dose of 100 ng VLPs. The enhanced cellular responses induced by the DNA prime-VLP boost were two- to three-fold greater than two DNA vaccinations. Moreover, a mixture of GagRT and GagTN VLPs also boosted antigen-specific CD8+ and CD4+ T-cell responses, while VLP vaccinations only induced predominantly robust Gag CD4+ T-cell responses. The results demonstrate the promising potential of these chimaeric VLPs as vaccine candidates against HIV-1.

## Findings

The importance of a cellular immune response against HIV-1 has been highlighted in several animal vaccine trials [1,2], with an abundance of evidence suggesting that an effective cellular immune response against HIV-1 is able to control and suppress viraemia during primary and chronic HIV infections, and to provide long-lasting protection [3-5]. Heterologous prime-boost vaccination has recently emerged as an effective means of enhancing T-cell responses [6-8], and current research suggests that HIV virus-like particles (VLPs) elicit a superior cellular immune response against HIV in animal models when used as a boost component in a prime-boost strategy [5,6]. In addition, previous studies have indicated the importance of including more than one HIV-1 proteins in a vaccine, due to the potential to induce broader and possibly more effective immune responses against HIV [9-11]. In this regard, Halsey *et al*. [12] showed that HIV-1 Pr55^Gag^-based chimaeric proteins with large C-terminal fusions both formed VLPs, and significantly enhanced T-cell responses elicited by a DNA vaccine to HIV-1 Gag and RT. The accessory proteins Tat, Nef and RT - which contain several prominent human cytotoxic T-lymphocyte (CTL) epitopes - are of particular interest in HIV vaccines: responses to Tat and Nef correlate with non-progression of HIV infections and possible protection [9], while RT-specific CTLs induce potent Th1 responses in mice, when administered in low doses [13].

This study investigates immune responses induced by chimaeric Gag VLPs incorporating RT and Tat-Nef sequences (GagRT and GagTN) as vaccine boost candidates to a DNA (pVRCgrttnC) priming vaccine expressing subtype C non-myristylated p6-deleted Gag, inactivated reverse transcriptase (RT), shuffled Tat (T) and inactivated Nef (N), as a polyprotein [14]. We further explored which combination of HIV-1 antigens in a VLP would best augment cellular immune responses induced by a complementary DNA vaccine, using DNA/VLP prime-boost vaccine regimens.

**Figure 1:**
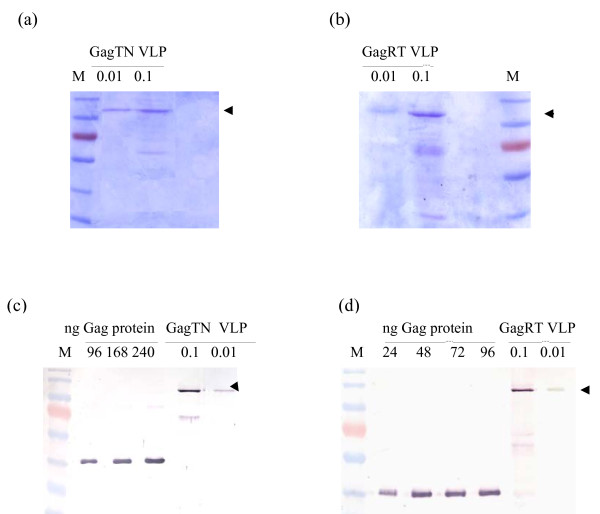
**Purity and quantification of Gag in VLP preparations**. Coomassie-stained SDS-PAGE gels of (a) GagTN VLP samples and (b) GagRT VLP samples indicating the purity of the samples. Western blots of (c) GagTN VLP samples and (d) GagRT VLP samples probed with anti-Gag p24 antibody. 0.1 and 0.01 indicate a 1:10 dilution and a 1:100 dilution, respectively, of VLPs loaded per lane. Western blots of Gag p17/p24 (p41) protein (24-240 ng) loaded per lane is indicated and the intensity of these bands was used to determine the Gag content of GagTN and GagRT VLP samples. M = molecular weight marker and arrowheads indicate the migration distance of the respective chimaeric VLPs.

The pVRCgrttnC DNA vaccine (1 mg/ml in PBS, manufactured by Aldevron, Fargo, ND, USA) is based on the pTHgrttnC vaccine described previously [14], but has the pVRC backbone (provided by the Vaccine Research Centre of the National Institutes of Health, Bethesda, Maryland, USA) in place of the pTH vector [15]. GagRT VLPs were expressed from the HIV-1 subtype C Gag precursor Pr55^Gag ^gene fused to the RT-encoding sequence from grttnC, and GagTN VLPs from a similar GagTatNef fusion [12]. Production of recombinant baculovirus-expressed GagRT and GagTN VLPs was optimized as described in Pillay *et al*. [16]. VLPs were purified from 2.5 L of *Sf*9 cell culture supernatants after 96 h incubation at 27°C. They were filtered through a 0.45 μm CFP-4-E-4MA polysulfone membrane capsule filter, and subsequently through a UFP-300-C-4MA polyethersulfone membrane (MWCO = 300 kDa (both Amersham)). Both filtration steps were necessary to remove cell debris and baculovirus contaminants from VLP samples. VLPs were pelleted by ultracentrifugation at 12 000 *g *for 60 min and re-suspended in PBS. Purity of the resulting VLPs was assessed using SDS-PAGE (Figure 1a and 1b). The presence of only the appropriate-sized protein bands indicated that no detectable contaminating material was present in the VLP samples. Endotoxin levels were < 0.125 endotoxin units per ml. Transmission electron microscopy (TEM) using a Zeiss S1109 electron microscope showed characteristic VLPs, albeit with a distribution of sizes [12]. Western blots probed with a 1:10 000 dilution of HIV-1 Gag p24 antibody (ARP432, NIBSC Centralised Facility for AIDS reagents, MRC, UK) and developed with goat anti-rabbit alkaline phosphatase conjugate (1:5 000; Sigma) and 5-bromo-4-chloro-3-indolyl-phosphate/nitroblue tetrazolium phosphatase substrate (BCIP/NBT; KPL) were used to quantify the Gag content of the VLP samples. The intensity of the Gag band in the VLP samples was compared to that of p17/p24-C standards (ARP695.2. FIT Biotech) using densitometry (Figure 1c and 1d).

Immunization of female BALB/c mice (8-10 weeks old, five per group; approved by the UCT Animal Research Ethics Committee, AEC No. 006-007) was by injection of 50 μl of the DNA dose (100 μg DNA/100 μl PBS) or 50 μl of the VLP dose (Gag protein dose in 100 μl PBS) into each hind leg muscle. Mice were primed with DNA on day 0 and boosted on day 28 with DNA or GagRT or GagTN or a mix of GagRT and GagTN. Initially, three doses of the chimaeric VLP boosts were evaluated to determine the optimal dose necessary for cellular immune response enhancement. These doses were 50 ng, 100 ng or 200 ng of Gag per 100 μl PBS, selected based on a previous DNA prime-VLP boost study [17]. Once determined, the optimal dose was used for the comparative mouse experiments. To test the immunogenicity of GagRT or GagTN alone, mice were left unprimed then vaccinated on day 28. Immune responses were detected on day 40.

Harvested spleens were pooled from a group of mice and isolated splenocytes were resuspended after erythrocyte lysis at 2 × 10^7 ^cells/ml R10 culture medium (RPMI with 10% heat inactivated FCS, Gibco, containing 15 mM β-mercaptoethanol, 100 U/ml penicillin, and 100 μg/ml streptomycin). Splenocytes were cultured in triplicate reactions with peptides (4 μg/ml) restricted to BALB/c mouse epitopes in Gag (CD8 peptide AMQMLKDTI; CD4(13) peptide NPPIPVGRIYKRWIILGLNK and CD4(17) peptide FRDYVDRFFKTLRAEQATQE), and RT (CD8 peptide VYYDPSKDLIA and CD4 peptide PKVKQWPLTEVKIKALTAI) in IFN-γ and IL-2 ELISPOT assays in triplicate (BD™Biosciences) [18]. Responses after subtraction of background in the absence of peptides are reported as mean spot forming units (sfu) ± standard deviation (SD) of the mean/10^6 ^splenocytes.

**Figure 2:**
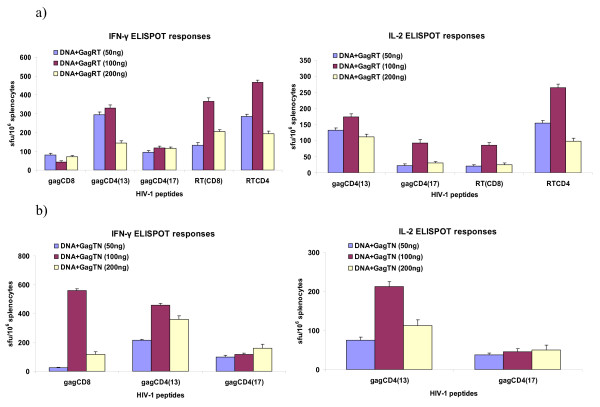
**Dose assessment for chimaeric VLP boost**. Three groups of five BALB/c mice were primed on day 0 with the DNA vaccine pVRCgrttnC and boosted with (a) GagRT or (b) GagTN on day 28. For all mouse groups spleens were harvested on day 40 and the splenocytes were used in IFN-γ ELISPOT and IL-2 ELISPOT assays with the indicated Gag and RT CD8 and CD4 peptides. Data represent results from one of three representative experiments.

Of the three doses assessed, the most prominent cellular immune responses were observed using a 100 ng VLP dose for the boost, with a two-fold differential in IFN-γ RT CD4 and CD8 responses for GagRT (Figure 2a), and a 10-fold increase in the IFN-γ Gag CD8 response for GagTN (Figure 2b). The overall stronger responses induced by the 100 ng dose indicated that this was the optimum dose for both GagRT and GagTN VLP boost vaccinations, and was used in subsequent experiments.

**Figure 3:**
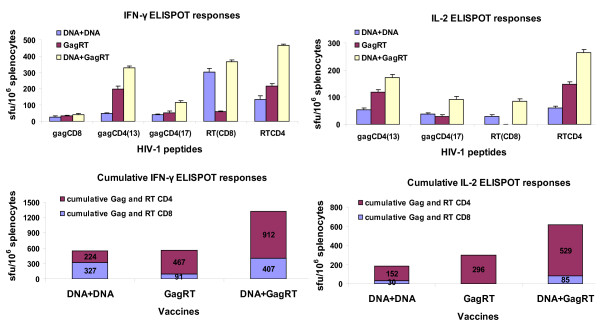
**Immunogenicity of GagRT VLPs**. BALB/c mice were primed on day 0 with the DNA vaccine pVRCgrttnC then boosted on day 28 with the DNA vaccine (DNA+DNA group) or GagRT VLPs (DNA+GagRT group). A further group was left unprimed then vaccinated on day 28 with GagRT VLPs. For all mouse groups spleens were harvested on day 40 and splenocytes used in IFN-γ ELISPOT and IL-2 ELISPOT assays with the indicated Gag and RT CD8 and CD4 peptides. Cumulative ELISPOT responses to the vaccines are shown and are to the sum of responses to the indicated peptides in the IFN-γ ELISPOT or IL-2 ELISPOT assays with the indicated Gag and RT CD8 and CD4 peptides. Data represent results from one of three representative experiments.

**Figure 4:**
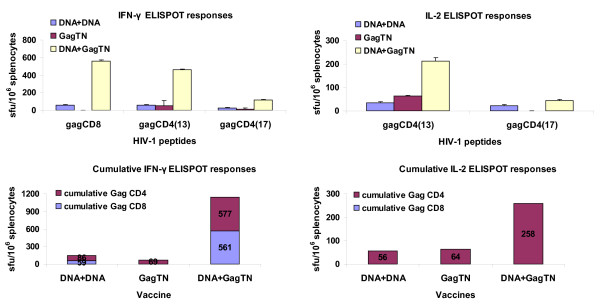
**BALB/c mice were primed on day 0 with the DNA vaccine pVRCgrttnC then boosted on day 28 with DNA (DNA+DNA group) or GagTN VLPs (DNA+GagTN group)**. A further group was left unprimed then vaccinated on day 28 with GagTN VLPs. For all mouse groups spleens were harvested on day 40 and splenocytes used in IFN-γ ELISPOT and IL-2 ELISPOT assays with the indicated Gag CD8 and CD4 peptides. Cumulative ELISPOT responses to the vaccines are shown and are to the sum of responses to the indicated peptides in the IFN-γ ELISPOT or IL-2 ELISPOT assays with the indicated Gag CD8 and CD4 peptides. Data represent results from one of three representative experiments.

After VLP-only vaccination with GagRT or GagTN, Gag- and RT- specific CD4+ cells contributed 84% to the cumulative IFN-γ ELISPOT response for GagRT, and 100% for GagTN. These VLPs also induced IL-2 Gag- and RT- specific responses (Figures 3 and 4). These responses were considerably enhanced using the heterologous prime-boost regimen.

The cumulative IFN-γ ELISPOT responses to a prime with DNA and boost with either GagRT or GagTN were greater by 2.4- and 16-fold respectively than two DNA vaccinations. The elicited responses after VLP boosts were predominantly from CD4+ cells (Figures 3 and 4). DNA prime/GagRT boost induced Gag and RT-specific IL-2 producing CD8 and CD4 cells while DNA prime/GagTN boost induced IL-2-producing Gag CD4 cells only. These IL-2 responses were 3.4- and 4.6-fold above that for two DNA vaccinations (Figures 3 and 4).

**Figure 5:**
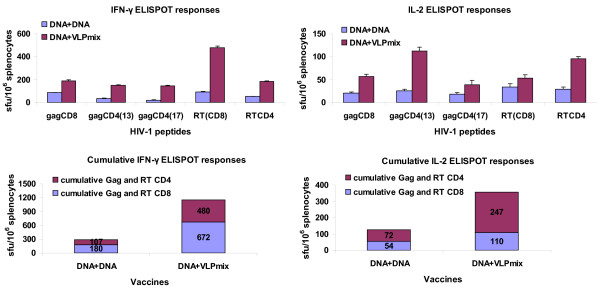
**Use of a mixture of GagRT and GagTN VLPs as vaccine boosts**. BALB/c mice were vaccinated with the DNA vaccine pVRCgrttnC on day 0 and day 28 (DNA+DNA group) or primed on day 0 with the DNA vaccine and boosted on day 28 with a mix of GagRT and GagTN (DNA+VLPmix group). For all mouse groups spleens were harvested on day 40 and splenocytes used in IFN-γ ELISPOT and IL-2 ELISPOT assays with the indicated Gag and RT CD8 and CD4 peptides. Data represent results from one of three representative experiments.

The DNA prime/mixed VLP boost vaccination increased the cumulative Gag- and RT-specific IFN-γ response relative to two DNA vaccinations 4-fold, and Gag- and RT-specific IL-2 response 2.8 fold. CD4+ cells contributed 41% to the cumulative IFN-γ and 31% to IL-2 ELISPOT responses, respectively (Figure 5).

In conclusion, this study extends and confirms results of our previous work in clearly showing that enhanced cellular immune responses against Gag and RT result when the chimaeric VLPs GagRT and GagTN are used in mice in a DNA vaccine prime/VLP boost vaccination regimen [12]. Novel results presented here include the induction of predominantly specific CD4+ cells producing IFN-γ and IL-2 by GagRT and GagTN vaccination alone. The lack of response to these VLPs in our previous study is probably due to the VLP dose being 1/5 of that used in this study [12]. Surprisingly, this 5-fold increase in GagRT dose did not increase the magnitude of the reported DNA prime/GagRT boost response, suggesting that only a low VLP dose is required for induction of recall cellular responses by primary vaccine-induced memory cells.

In general, the magnitude of responses induced were better using a single VLP boost, than with the mixed VLP boost. This is possibly related to an antigen dose effect, where above the optimum dose, antigen-specific cellular immune responses decline [19]. The results of the boost dose assessment (Figure 2) support this hypothesis, as the 200 ng VLP boost (which is equivalent to the mixed VLP dose administered) induced much lower responses than the 100 ng dose. That being said, the mixed VLP boost was able to elicit more proportionate, cumulative CD4+ and CD8+ specific T-cell responses. Expansion of specific CD4+ cells in response to vaccination is important in that these cells play a key role in activation of both B cells and CD8+ cells, and in controlling CTL responses [20], and are linked to the control of HIV-1 infection and replication [21,22]. However, it is the CD8+ cell response that is mainly responsible for controlling the viral infection and suppressing viraemia in HIV-infected individuals [23]. Hence, the CD4+ and CD8+ cell boost observed with vaccination of a VLP mix, and the finding that HIV-specific CD4+ and CD8+ cells produce IFN-γ and IL-2, are of strong significance. Collectively, the data shown here demonstrates that chimaeric VLP boosts such as these are a promising option for future HIV-1 vaccine studies.

## Competing interests

The authors declare that they have no competing interests.

## Authors' contributions

SP produced and purified the chimaeric VLPs, participated in performing the ELISPOT assays, analysed the immunology data with ES, and drafted the manuscript. ES and her group were responsible for carrying out the mouse experiments and performing the ELISPOT assays. AM and EPR were involved in supervision of the work, critically revising the manuscript for important intellectual content, and together with A-LW, conceived of the study, and participated in its design and coordination. All authors read and approved the final manuscript.
